# Bone mineral density loss in postmenopausal onset rheumatoid arthritis is not greater than premenopausal onset disease

**Published:** 2014

**Authors:** Behzad Heidari, Parham Heidari

**Affiliations:** 1 Mobility impairment research center, Babol University of Medical Sciences, Babol, Iran.; 2 Department of Medicine Division of Rheumatology Ayatollah Rouhani Hospital, Babol University of Medical Sciences, Babol, Iran.; 3 Avina Clinic, No, 5. Jordan ST. Tehran, Iran.

**Keywords:** Rheumatoid arthritis, Age of onset, Postmenopausal, Premenopausal, Osteoporosis, Bone mineral density

## Abstract

***Background: ***Postmenopausal onset rheumatoid arthritis (post-RA) is expected to have greater bone mineral density (BMD) loss than premenopauasal onset (pre-RA) due to estrogen deficiency and aging. This study aimed to compare the BMD status of the two RA groups with age-matched non-RA controls.

***Methods:*** The patients with RA on follow-up examination were stratified according to age of onset. Femoral neck and lumbar spine BMD (FN-BMD and LS-BMD) were assessed by DXA method. The patients of the two groups were compared with non-RA controls in regard to BMD gr/cm^2 ^and the risk of osteoporosis (OP).

***Results:*** Forty-eight post-RA and 94 pre-RA were compared with 31 and 57 age-matched controls. FN-BMD gr/cm^2 ^and LS-BMD gr/cm^2^ in both groups of RA was significantly lower than the controls (P=0.001 for all). In post-RA, FN-BMDgr/cm^2 ^was 16% lower than controls versus 21% in pre-RA, whereas, LS-BMD reductions were 5% and 12%, respectively (P=NS). FN-OP was observed in 32(68%) and 9 (29%) post-RA and controls (P=0.001) versus 29 (30.8%) and 4 (7%) pre-RA and controls, respectively (P=0.001). Corresponding percentages for LS-OP in post-RA and controls were (37.5% vs 35.5%, P=0.52) and in pre-RA and controls were (21.3% vs 3.5%, P=0.002), respectively. Risk of osteoporosis at either measurement sites of FN or LS in post-RA increased by the adjusted odds of 1.54(95% CI, 0.60-3.9, P=0.36) and in pre-RA by the adjusted odds of 5 (95% CI, 1.78-14.5, P=0.002), respectively.

***Conclusion: ***These findings indicate that BMD loss in post-RA is not greater than pre-RA as expected. It is possible that estrogen deficiency by modulating immunologic reactions compensates the negative effects of estrogen deprivation on bone mass in post-RA patients.

Rheumatoid arthritis (RA) is characterized by progressive inflammatory process directing to structural joint disease and bone loss. In patients with RA, several factors like persistent inflammation, disease duration, and limited physical activity due to joint injury, disease activity, and treatment with corticosteroid are associated with bone loss and ostoporosis (1-6). RA is more prevalent in females and its peak incidence coincides with perimenopausal period suggesting a relationship between sex hormones and development of RA (7-10). Early menopause is a risk factor for the development of RA (11).

Nevertheless, the administration of sex hormones during postmenopausal stage showed no protective effect on the development of RA (12). Sex hormones may modulate autoimmune disorders or result in disease onset or its perpetuation (13). In normal conditions, estrogens exert beneficial effect on bone mass and therefore, estrogen deficiency may provide additional risk factor for increasing bone loss in postmenopausal RA as compared with premenopausal women (7, 14). The age at onset of RA may also affect the clinical pictures, disease severity, cytokines distribution and prognosis of RA (15). The patients with elderly-onset RA seem to have milder disease and lower radiologic progression as compared with young-onset RA (16). Therefore, in comparable patients, women with postmenopausal versus premenopausal onset RA may be differently exposed to hormonal variations. Thus, the pattern of bone loss and bone mass outcome may differ according to the age of onset of RA.

The most important cause of disability in RA, is the occurrence of vertebral and non-vertebral fractures (17). The possibility of these complications is greater in postmenopausal RA due to estrogen deprivation which causes marked stimulation of bone resorption and rapid bone loss. Nevertheless, the bone mass status of postmenopausal onset RA has not been compared with premenopausal RA. This issue is important because postmenopausal RA seems to be at greater risk of bone loss and awareness to associated factors of ostoporosis facilitates preventive measures. Thus, the present study was designed to determine and compare bone mass status in postmenopausal and premenopausal RA by comparison to age-matched non- RA controls.

## Methods

The diagnosis of RA was confirmed by the American College of Rheumatology (ACR) revised criteria (18). All patients were recruited among patients presented for follow-up examination to an outpatient rheumatology clinic. Data were collected for age, menopausal status, interview age of onset, duration of disease, duration of treatment, menopausal duration, clinical examination and review of medical records. All patients were treated similarly with at least one disease modifying anti-rheumatic drug (DMARD), such as methotrexate (MTX) and hydroxychloroquine, with or without low-dose prednisolone (<7.5mg/daily). The dosage of MTX was adjusted according to clinical response and the laboratory test results with the goal of disease remission. Exclusion criteria were the presence of chronic systemic and inflammatory diseases and conditions which might affect physical activities and bone metabolism. BMD at the femoral neck (FN-BMD) and lumbar spine (L2-L4, LS-BMD) was performed by dual energy x-ray absorptiometry (DEXA) after median disease duration of 3.8 (0.12-200 years. Osteoporosis was confirmed according to the WHO criteria (19) defined as BMD values of 2.5 SD or more below the mean value for young adults (T-score < -2.5). 

In addition, the frequency of osteoporosis at either measurement sites of FN or LS were determined based on the lowest T -score (T-score ≤ - 2.5) of measured skeletal sites as proposed by the International Society for Clinical Densitometry (one diagnostic category) (20). 

The primary objective of this study was to compare BMD status in postmenopausal onset RA (post -RA) versus premenopausal onset RA (pre -RA) by comparison of the two groups of RA with aged- matched postmenopausal and premenopausal non- RA controls. The secondary objective was to determine and compare the prevalence and the risk of osteoporosis in both groups of patients. In statistical analysis, the patients with RA were stratified to post-RA and pre-RA according to age of disease onset with regard to menopausal period. Age-matched non- RA controls were selected among patients presented for bone densitometry at the same clinic. A similar exclusion criteria was applied for the control group. In data analysis, the patient groups were compared with age-matched control groups regarding to the proportion of osteoporosis at FN and /or LS. Mean BMD gr/cm^ 2^ differences from controls in each RA group were determined and compared. In addition, the risk of osteoporosis in each RA group was determined by the calculation of odds ratio (OR) after adjustment for age, menopausal duration with corresponding 95% confidence interval (95%CI) using multiple logistic regression analysis. SPSS software Version 18 was used for data analysis. The proposal of this study was approved by the Ethics Committee of Babol University of Medical Sciences, Babol, Iran.

## Results

A total of 142 women with RA were studied. Forty-eight post- RA and 94 pre- RA were analyzed. The two groups of patients were similar regarding to duration of RA, treatment regimen, rheumatoid factor and anti-cyclic citrullinated antibody seropositvity and (table 1). The patients of the two groups were compared with 31 postmenopausal and 57 premenopausal controls, respectively. As shown in table 2, the FN-BMD gr/cm^2^ and LS -BMD gr/cm^2^ in both groups of RA was significantly lower than the controls (P=0.001 for all). In post- RA, the mean FN-BMD gr/cm^2 ^was 16% lower than postmenopausal controls and in pre-RA, FN-BMD gr/cm^ 2 ^was 21% lower than premenopausal controls. The corresponding values for LS-BMD gr/cm^ 2 ^were 5% and 12% respectively (table 2). Osteoporosis at either FN or LS in post- RA and age-matched controls was observed in 32(68%) and 17 (54.8%) patients, respectively (P=0.20). In pre- RA and age-matched controls, osteoporosis was found in 34 (36.2%) and 5 (8.8%) patients, respectively (P=0.001). The risk of osteoporosis at either FN or LS in post-RA and pre-RA increased by adjusted OR of 1.54(95% CI, 0.60-3.9, p=0.36) and 5 (95% CI, 1.78-14.5, P=0.002), respectively (table 3). In post- RA group, the odds of osteoporosis increased 2.2% for each year (95% CI, - 6% - 11%, P= 0.60) and in pre- RA by 14.5% per year CI (95% CI, 6.9% - 22.6%, P=0.001). The odds of osteoporosis increased significantly by aging (P=0.02) whereas, in post-RA there was no relationship between odds of osteoporosis with aging. 

**Table 1:** Characteristcs of rheumatoid arthritis patients according to age of onset respect to menopausal state

**Patients characteristics**	**Postmenopausal onset** **No=47**	**Premenopausal Onset** **No=94**	**P values**
Age, mean±SD	64±7.1	44.9±10	
Disease duration, years median	3 (0.16-20)	4.4(0.12-20)	0.39
Duration of treatment, year Media	2.5 (0.12-6.5)	3 (0.12-12)	0.87
Anti-CCP positivity, no (%)	37 (78.7)	74 (78.7)	0.60
RF positivity, no (%)	35 (74)	63 (67)	0.32
Methotrexate therapy no (%)	14 (29.7)	35 (37.2)	0.33
Low-dose prednisolone therapy, no (%)	35 (74)	78 (83)	0.29

Anti-CCP=Anti cyclic citrullinated peptide antibody

RF= Rheumatoid factor

**Table 2:** Bone mineral density at the femoral neck FN-BMD gr/cm^2^) and lumbar spine (LS-BMD gr/cm^2^) in patients with rheumatoid arthritis (RA) according to age of onset of disease during postmenopausal stage (Post-RA) or premenopausal stage (Pre-RA) compared with age-matched postmenopausal or premenopausal non –RA controls

**BMD measuremen sites**	**Postmenopausal period**			**Premenopausal period**		
	**RA** **N=47**	**Controls** **N=31**	**Mean differences**	**RA** **n-94**	**Controls** **N=57**	**Mean differences**
FN-BMD gr/cm2	0.64±0.15	0.76±0.12	-16% P=0.001	0.743±0.13	0.91±0.14	-21% P=0.001
LS-BMD gr/cm2	0.74±0.13	0.78±0.11	-5% P=0.17	0.85±0.18	0.97±0.14	-12% P=0.001

¥ Mean difference between patients with RA and age-matched controls

**Table 3:** Frequency of osteoporosis (OP) at the femoral neck (FN-OP) and lumbar spine (LS-OP) or at either FN or LS in postmenopausal and premenopausal onset rheumatoid arthritis (RA) versus postmenopausal and premenopausal non-RA controls

**BMD measurement sites**	**Postmenopausal period**			**Premenopausal period**		
	**RA** **N=47**	**Controls** **N=31**	**P values**	**RA** **n-94**	**Controls** **N=57**	**P values**
FN-OP	32 (68)	9 (29)	0.001	29 (30.8)	4 (7)	0.001
LS-OP	18 (38.3)	11 (35.5)	0.52	20 (21.2)	2 (3.5)	0.002
FN-OP /or LS-OP	32 (68)	17 (54.8)	0.2	34 (36.1)	5 (8.8)	0.001
OR(95%CIl)҂	1.54 (0.60-3.9)	1	0.36	5 (1.78-14.5)	1	0.002

҂ Multiple logistic regression analysis

## Discussion

The findings of this study indicated that, in patients with RA, bone mass at the FN and LS was significantly lower than age-matched controls regardless of the age of disease onset. In pre- RA patients with median disease duration of 4 years, the risk of osteoporosis at the FN and/ LS increased significantly by odds of 5, whereas, in post-RA, the risk of osteoporosis did not increase to a statistically significant level after a median disease duration of 3 years. 

Furthermore, there was a significant positive relationship between the odds of osteoporosis and aging in pre- RA but not in post-RA. Additionally, as compared with age-matched controls, bone loss at both measurement sites in post- RA was non-significantly lower than pre- RA. With regard to similarities in patient characteristics, treatment regimen between the two groups of RA, these findings indicate milder disease in the post- RA group with much lower bone loss compared to pre -RA patients. The results of this study are consistent with the findings of many studies which compared RA patients according to age of onset (21-24).

The age of onset of RA impresses the clinical and laboratory features, joint score and HLA-DRB1 alleles expression (15). The clinical characteristics and pattern of inflammatory cytokines in the elderly-onset RA differ from the young onset RA (21). In one study, the patients with average age onset of 68±4.6 years had milder disease with lower frequency of some autoantibodies compared with those age of onset at 42.2±10.4 years. In the latter study, among the patients with similar age, those younger-age onset had more severe RA with higher disease activity, lower functional capacity and lower remission rate when compared with older-age onset group (22). Elderly-onset RA is milder and more common in males (16). Additionaly, HLA-DRB1 which seems to be protective against anti-CCP positivity is more frequent in patients with elderly-onset disease (25). In one study, elderly-onset RA required less biologic drugs or DMARDs compared to young-onset RA even with comparable disease severity (24).Therefore, lower bone loss in post-RA patients of this study can be partly explained by older-age of onset.

Another possibility of BMD preservation in post- RA group may be attributed to weight gaining by aging (26). Furthermore, the high prevalence of osteoarthritis in elderly subjects (27, 28) may result in falsely elevated LS-BMD (10).There is a positive relation between BMI and FN-BMD particularly in women (1). Estrogen, in addition to anti-resorptive characteristics exerts some immunologic activities (13, 24, 29-31). Lack of estrogen in post-RA is expected to be associated with greater bone resorption and bone loss (29, 31), On the other hand, the presence of estrogen in pre-RA patients increases cell growth and enhances immune responses through stimulating B cells and inhibiting T cells and macrophages in a dose-dependent manner (24, 31). Furthermore, estrogen increases TNF-a - induced MAP-3 and thus, results in RA progression (32).

Hence, estrogen-related immunologic reactions impose pre- RA patients to greater risk of bone loss and generalized osteoporosis (9) whereas, in estrogen deficient post – RA, this reaction is not expected to occur.

These observations indicate that the negative effect of estrogen deficiency in post-RA may be in part compensated or modulated by age of disease onset or immunologic consequence of estrogen deprivation (13, 30, 31). The results of this study should be considered with limitations. Many factors like disease activity, drug dosage, corticosteroid therapy, level of physical activities, number of parity and treatment may affect the results. In particular, the impact of treatment on disease activity may exert beneficial effect on BMD and lead to BMD changes (2, 4, 5, 33).

Since both groups of patients and controls were recruited from a single population with similar lifestyle and ethnic characteristics, thus, the results are expected to be less confounded. In particular, the two groups of patients were similar in regard to disease duration, RF and anti- CCP positively, corticosteroid and DMARDs therapy. Another limitation is related to study design which was not performed prospectively to assess the magnitude of bone loss compared to baseline value. Nevertheless, the comparison of the two groups of RA with age-matched controls, can display reliable data for comparison. 

 In conclusion, the results of this study indicated that in spite of estrogen deficiency during postmenopausal period, the magnitude of bone loss in post-RA is not greater than pre-RA as compared with age-matched controls. It is possible that the age of onset or estrogen-related changes on immune response may attenuate disease course and results in milder RA with lower expected bone loss.

## Notes

**Funding: **Self - Funded.
